# Intraoperative Fat Embolism Syndrome Causing Acute Right Ventricular Failure Successfully Treated With Veno-Arterial Extracorporeal Membrane Oxygenation: A Case Report

**DOI:** 10.7759/cureus.102383

**Published:** 2026-01-27

**Authors:** Shogo Mabuchi, Ikuko Miyawaki

**Affiliations:** 1 Critical Care Medicine, National Cerebral and Cardiovascular Center, Osaka, JPN; 2 Anesthesiology, Kobe City Medical Center General Hospital, Kobe, JPN

**Keywords:** acute respiratory distress syndrome, extracorporeal membrane oxygenation, fat embolism syndrome, mechanical circulatory support, pulmonary hypertension, right ventricular failure

## Abstract

Fat embolism syndrome (FES) is a rare but potentially fatal complication of orthopedic surgery. Although extracorporeal support has been reported in trauma-related FES, the use of veno-arterial extracorporeal membrane oxygenation (V-A ECMO) for FES during elective arthroplasty has not been described. We report a case of a 67-year-old woman undergoing revision total hip arthroplasty. During the procedure, she developed profound hypoxemia and refractory hypotension following an iatrogenic femoral fracture prior to cement fixation. Despite fluid resuscitation and vasopressor and inotropic therapy, cardiovascular collapse progressed. The clinical course was consistent with FES complicated by acute right ventricular failure and pulmonary hypertension, and V-A ECMO was initiated for circulatory support. The patient was successfully liberated from ECMO on postoperative day 4 and discharged home without major complications. This case illustrates that fulminant intraoperative FES can cause abrupt right ventricular failure even during elective arthroplasty and suggests that early recognition and timely initiation of V-A ECMO may be lifesaving.

## Introduction

Fat embolism syndrome (FES) is characterized by the classical triad of symptoms, including respiratory distress, rash, and neurological abnormality [1]. While this syndrome is most commonly associated with long-bone trauma, it can also arise as a rare complication of orthopedic procedures involving intramedullary manipulation [2]. Although the clinical course is often self-limiting, severe FES can be life-threatening. Successful resuscitation with extracorporeal circulation has been reported in a few clinical cases of critical post-traumatic FES; however, to our knowledge, the use of veno-arterial extracorporeal membrane oxygenation (V-A ECMO) for FES with complete cardiovascular collapse occurring in an elective orthopedic procedure has not been previously described [3]. Here, we report a case of FES that ultimately necessitated V-A ECMO.

## Case presentation

A 67-year-old woman with a past medical history of subarachnoid hemorrhage, symptomatic seizures, a ventriculoperitoneal shunt for normal pressure hydrocephalus, and aspiration pneumonia presented for revision total hip arthroplasty (THA) with cementation because of the malfunction of a prosthesis implanted two years earlier. She was 145.1 cm tall and weighed 52.7 kg, with a body mass index of 25.03 kg/m². Preoperative evaluation revealed no notable findings except for an elevated D-dimer level of 3.27 μg/mL. Lower extremity duplex ultrasonography showed no evidence of thrombosis. Electrocardiography (ECG) and chest radiography (CXR) were unremarkable. Surgery was planned under general anesthesia with a peripheral nerve block.

During the operation, the surgical team encountered difficulties with femoral stem extraction, which led to fractures of the greater trochanter and femoral shaft. Subsequently, before cement fixation, the patient developed progressive hypoxemia and hypotension. The hypotension was refractory to several bolus doses of phenylephrine (50 μg per bolus) and colloid infusion, necessitating continuous phenylephrine infusion at 0.15 μg/kg/min. These measures failed to stabilize her vital signs, prompting administration of norepinephrine boluses followed by continuous infusion, which was also ineffective. At this point, the surgical procedure was aborted.

Initially, the patient’s hemodynamic instability was attributed to hemorrhage from the intrapelvic venous plexus, and massive fluid resuscitation was initiated. However, after undraping the patient, jugular venous distension was observed, raising suspicion for pulmonary embolism. Contrast-enhanced computed tomography was performed for diagnostic evaluation. The scan showed no evidence of hemorrhage or thrombosis but demonstrated bilateral infiltrates, ground-glass opacities, and intralobular septal thickening, suggestive of pulmonary edema. Based on the overall clinical assessment, FES was considered.

**Figure 1:**
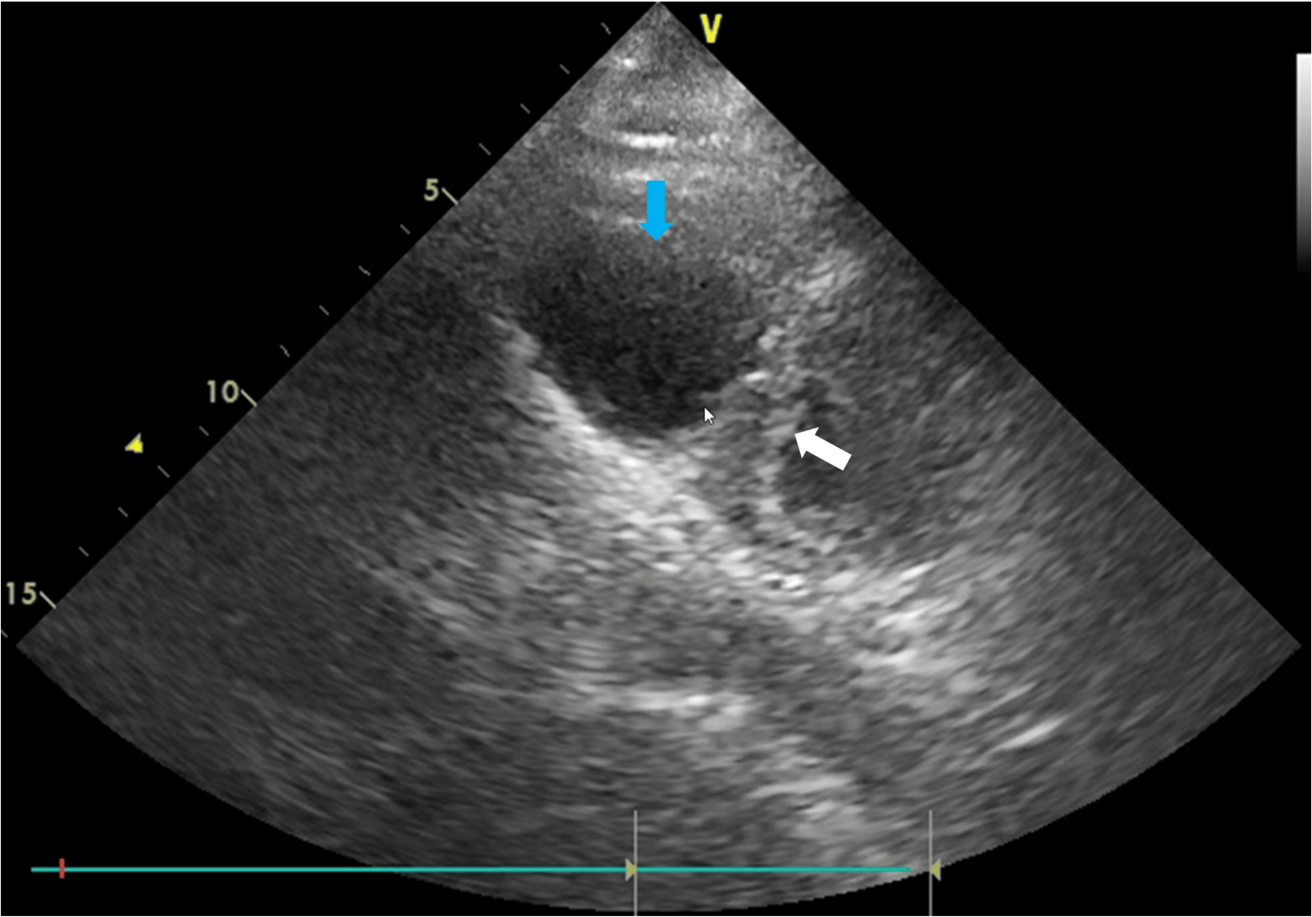
Transthoracic echocardiography performed after admission to the intensive care unit Parasternal short-axis view from transthoracic echocardiography performed after admission to the intensive care unit, showing severe right ventricular dilatation (blue arrow) with interventricular septal flattening toward the left ventricle (white arrow), findings consistent with acute right ventricular overload.

Postoperatively, in the intensive care unit, transthoracic echocardiography (TTE), ECG, and CXR were performed. TTE revealed severe right ventricular (RV) dilatation with septal flattening toward the left ventricle (Figure 1).

**Figure 2:**
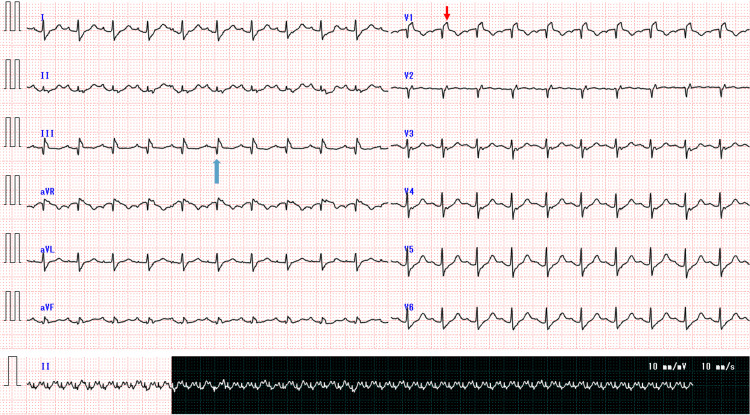
Electrocardiogram demonstrating acute right heart strain Electrocardiogram showing new-onset right bundle branch block with a prominent terminal R′ wave in lead V1 (red arrow) and a Q wave in lead III (blue arrow), findings consistent with acute right ventricular strain.

ECG showed new-onset complete right bundle branch block and a pathological Q wave in lead III (Figure 2).

**Figure 3:**
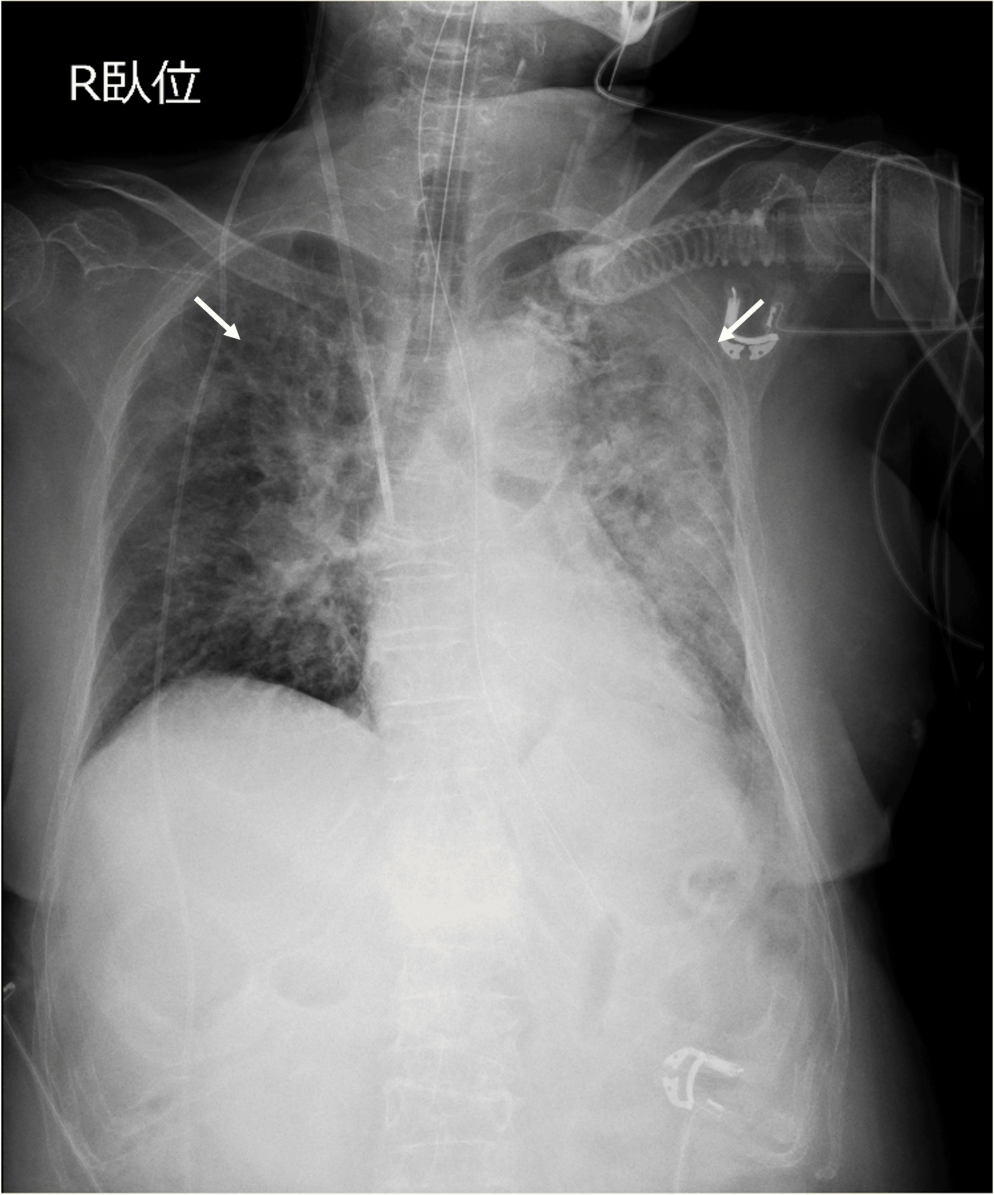
Chest radiograph after admission to the intensive care unit Chest radiograph shows bilateral diffuse pulmonary infiltrates (white arrows)

CXR demonstrated bilateral infiltrates (Figure 3).

A pulmonary artery catheter (PAC) was placed for hemodynamic monitoring and revealed pulmonary hypertension with a markedly narrow pulmonary pulse pressure of 3 mmHg (pulmonary artery pressure, 33/30 mmHg). Based on these findings, a diagnosis of acute respiratory distress syndrome and cardiogenic shock due to RV failure associated with FES was made.

At the time of admission to the intensive care unit, the patient was mechanically ventilated under volume-controlled ventilation with a tidal volume of 6 mL/kg, fraction of inspired oxygen (FiO₂) of 0.60, and positive end-expiratory pressure (PEEP) of 9 cmH₂O. Despite escalation of ventilatory settings to FiO₂ 0.95 and PEEP 10 cmH₂O, the patient’s condition continued to deteriorate under high-dose vasoactive support, including noradrenaline 0.47 μg/kg/min, dobutamine 9.49 μg/kg/min, and adrenaline 0.16 μg/kg/min. Consequently, V-A ECMO was initiated, along with continuous kidney replacement therapy (CKRT) for volume management. V-A ECMO was established via right femoral arterial and venous cannulation without procedural complications.

Following initiation of V-A ECMO, pulmonary hypertension and circulatory instability showed gradual improvement over time. From postoperative day one to three, pulmonary artery pressures decreased progressively with volume management using CKRT, vasoactive requirements were reduced, and ECMO flow was maintained at 1.6-2.0 L/min.

On postoperative day three, a V-A ECMO liberation trial was attempted as pharmacologic hemodynamic support was tapered. With dobutamine at 3.80 μg/kg/min and adrenaline at 0.03 μg/kg/min, ECMO flow was reduced from 1.9 L/min to 0.9 L/min. During this trial, hemodynamic parameters deteriorated: mixed venous oxygen saturation decreased from 70% to 65%, pulmonary artery pressure increased from 28/22 mmHg to 33/27 mmHg, central venous pressure increased from 12 to 13 mmHg, and systolic arterial blood pressure decreased from 105 to 95 mmHg. Given the persistently low pulmonary pulse pressure and insufficient improvement in pulmonary hypertension, the trial was discontinued.

On postoperative day four, the patient’s hemodynamic status further improved, and a second weaning attempt was performed. Clamping the return cannula resulted in minimal hemodynamic impact, with a slight decrease in mixed venous oxygen saturation from 68% to 64%. Cardiac index increased from 2.3 to 2.6 L/min/m², pulmonary artery pressure from 44/19 mmHg to 51/22 mmHg, central venous pressure from 8 to 9 mmHg, and arterial blood pressure changed from 106/65 mmHg to 93/53 mmHg. Although pulmonary vascular resistance remained elevated, no interventricular septal shift was detected on TTE, and oxygenation remained adequate. The patient was successfully liberated from V-A ECMO.

The patient was extubated on postoperative day eight, and CKRT was discontinued. Her postoperative course was complicated by a surgical site infection requiring removal of the femoral stem. After a prolonged recovery period, a final revision THA was performed on postoperative day 39. The patient was discharged home on postoperative day 53 without major complications.

## Discussion

We report the first case of fulminant FES with cardiovascular collapse in elective orthopedic surgery unrelated to pre-existing trauma, successfully rescued with V-A ECMO. This case highlights the pathophysiology of FES-induced right ventricular failure, the challenge of intraoperative diagnosis, and the critical decision-making surrounding advanced mechanical support.

The "classical triad" of FES is petechial rash, neurological impairment, and respiratory distress, with respiratory failure being the most common manifestation. Although hypoxemia is observed in 90% of FES cases [4], circulatory collapse requiring extracorporeal resuscitation is rare. In such critical scenarios, veno-venous ECMO (V-V ECMO) is typically preferred over V-A ECMO.

The most common cause of FES is trauma [5]. Two theories have been proposed for the mechanism of FES. The first is the mechanical theory. This theory posits that a fracture disrupts intramedullary fat, which then enters the circulation and can create mechanical emboli. Fat can be involved in ruptured veins because calcified tubules that contain the veins keep their ends open after rupture, and the negative venous pressure draws free fat globules into them.

The other theory is the biochemical theory. According to this theory, embolized tissues provoke an inflammatory response where proinflammatory cytokines such as interleukin (IL)-1, IL-6, and tumor necrosis factor-α can cause ARDS as seen in this case. Severe hypoxia due to ARDS constricts pulmonary vasculature and increases pulmonary vascular resistance (PVR) [6]. Since pulmonary circulation is based on low-resistance/low-pressure physiology, elevated PVR imposes a significant afterload on the RV, leading to RV failure in some cases [7]. It is of note that RV failure develops in up to 25% of cases of ARDS [8,9].

Diagnosis of FES is challenging because of the lack of a gold standard. Several criteria and laboratory findings have been proposed and may be of use but are not definitive [1,10,11]. An important differential diagnosis for intraoperative cardiopulmonary collapse during cemented arthroplasty is bone cement implantation syndrome (BCIS), which can present with a clinical picture similar to FES. However, several factors in our case pointed towards FES. The patient's progressive hypoxemia and hemodynamic instability began to develop coinciding with the femoral fracture, which occurred prior to the cementation process. This iatrogenic long-bone fracture served as a potent trigger for the massive release of intramedullary contents. Furthermore, contrast-enhanced CT imaging excluded other major embolic sources, and the patient’s overall clinical presentation fulfilled the diagnostic criteria for FES.

FES is a potential complication of arthroplasty, particularly during intramedullary instrumentation, where intramedullary pressure increases, causing fat to enter the circulation [2]. However, respiratory and circulatory failure associated with arthroplasty is rare [12]. In fact, fat embolism from arthroplasty requiring ECMO has not yet been reported.

While some risk factors for trauma-induced FES are known, such as long-bone fractures, open fractures, and the time from injury to surgery [13], the risk factors for FES during elective orthopedic surgery remain uncertain. Although the fixation method is theoretically associated with FES [14], past studies produced controversial results [15,16].

The initial management of ARDS-induced RV failure typically includes optimization of the patient’s volume status, pharmacologic support of RV contractility, and reduction of RV afterload. Nonetheless, in cases of failure to achieve hemodynamic stability despite maximal conventional therapy, extracorporeal resuscitation becomes necessary.

Regarding extracorporeal support, the choice between V-V and V-A ECMO was the critical therapeutic decision. In cases of ARDS-induced RV failure, V-V ECMO can theoretically be sufficient in reversing hypoxemia, which in turn can alleviate hypoxic pulmonary vasoconstriction and unload the RV [17]. This can be the case in the presence of hemodynamic compromise [18]. However, these studies suggest that patients with acute cor pulmonale successfully treated with V-V ECMO have a higher cardiac index than our patient. Therefore, in the face of profound cardiovascular collapse with high-dose multi-agent vasopressor and inotropic support, relying solely on the indirect and gradual improvement of PVR due to V-V ECMO would have been insufficient to restore vital organ perfusion. As such, V-A ECMO was the only viable option to provide immediate, direct circulatory support in our case.

While V-A ECMO has been reported in fulminant FES, previously published cases occurred in the setting of pre-existing traumatic long-bone fractures [19,20]. By contrast, the present case developed during elective orthopedic surgery in a patient without any fracture prior to surgery, with fulminant FES triggered by an intraoperative iatrogenic fracture. This distinction highlights that catastrophic FES with acute right ventricular failure can occur even during planned procedures in patients initially considered to be at low risk.

## Conclusions

Fulminant FES can occur during elective orthopedic surgery without pre-existing trauma and may progress to acute right ventricular failure with refractory cardiovascular collapse. Because the diagnosis is often challenging in the intraoperative setting, clinicians must maintain a high index of suspicion when unexpected hypoxemia and hemodynamic instability develop.

When conventional supportive measures fail, early consideration of advanced mechanical circulatory support is essential. This case highlights that V-A ECMO can serve as a lifesaving option in selected patients with FES complicated by severe right ventricular failure, emphasizing the importance of timely and decisive intervention even in planned surgical procedures.
